# Therapeutic advances in spinal muscular atrophy: a review of clinical, safety, and economic considerations

**DOI:** 10.3389/fphar.2026.1793581

**Published:** 2026-09-14

**Authors:** Andrej Belančić, Elvira Meni Maria Gkrinia, Yun Wah Lam, Dinko Vitezić

**Affiliations:** 1 Department of Basic and Clinical Pharmacology and Toxicology, University of Rijeka, Faculty of Medicine, Rijeka, Croatia; 2 Independent Researcher, Athens, Greece; 3 Department of Health Sciences, School of Nursing and Health Sciences, Hong Kong Metropolitan University, Hong Kong, Hong Kong SAR, China

**Keywords:** disease-modifying therapies, nusinersen, onasemnogene abeparvovec, risdiplam, spinal muscular atrophy

## Abstract

Spinal muscular atrophy (SMA) is a genetic neuromuscular disorder caused by insufficient levels of survival motor neuron (SMN) protein due to mutations in the *SMN1* gene, leading to progressive degeneration of lower motor neurons in the spinal cord. In this comprehensive narrative review, we extensively summarise the clinical efficacy and real-world effectiveness of approved disease-modifying therapies, including nusinersen, onasemnogene abeparvovec, and risdiplam, underscoring significant improvements in survival, motor milestones, and quality of life, particularly with early treatment initiation. Safety profiles and post-marketing surveillance data are examined, revealing manageable adverse events aligned with clinical trial findings. Economic evaluations are also discussed, illustrating the high costs associated with SMA therapies and the challenges in cost-effectiveness under conventional thresholds, while noting the potential advantages of some treatments in specific contexts. In the review, we explore the promising pipeline of investigational therapies targeting both SMN-dependent and independent mechanisms such as muscle enhancers and neuromuscular junction modifiers, reflecting evolving multidimensional treatment strategies. Future directions emphasise early diagnosis through newborn screening, optimised treatment regimens, combination therapies, biomarker-guided personalised approaches, and comprehensive multidisciplinary care. Lessons from SMA provide a valuable framework for rare-disease management, highlighting the importance of mechanistic insights, early intervention, collaborative research, patient advocacy, and innovative health technology assessment to improve access and outcomes. Our aim is for the review to guide clinical practice, research priorities, and health policy by synthesising contemporary advances and challenges in SMA treatment and care.

## 1 Disease background and brief overview of approved medicines for SMA

Spinal muscular atrophy (SMA) is a rare autosomal recessive neuromuscular disorder caused by homozygous deletions or mutations in the *survival motor neuron 1* (*SMN1*) gene, leading to insufficient survival motor neuron (SMN) protein and progressive degeneration of lower motor neurons (Darras, 2015; Verhaart et al., 2017). Clinically, SMA presents with symmetric muscle weakness, hypotonia, and atrophy, with a spectrum ranging from severe prenatal or infantile-onset forms (type 0–1) to adult-onset presentations (type 4) characterised by mild proximal weakness (Chen, 2020; Talbot and Tizzano, 2017; Tizzano and Finkel, 2017). Disease severity is strongly influenced by *SMN2* copy number: although *SMN2* is a nearly identical paralog of *SMN1*, a C → T transition in exon 7 promotes exon 7 skipping, mainly leading to unstable SMND7 protein and only limited full-length SMN protein. Other modifiers, including *PLS3*, *NCALD*, and pathways involved in RNA processing and axonal biology, may further modulate the phenotype. Historically, SMA was a leading genetic cause of infant mortality, but newborn-screening and disease-modifying therapies (DMTs) now enable earlier diagnosis and treatment before significant motor neuron loss occurs (Chen, 2020; Talbot and Tizzano, 2017; Hassan et al., 2020; Tizzano and Finkel, 2017; Farrar et al., 2020).

The past decade has witnessed a paradigm shift in SMA management with the approval of three DMTs: nusinersen, an intrathecal antisense oligonucleotide that modifies *SMN2* splicing (Finkel et al., 2017; Mercuri et al., 2018); onasemnogene abeparvovec, an intravenous adeno-associated viral vector that provides functional copies of *SMN1* to motor neurons (Mendell et al., 2017; Day et al., 2021; Mercuri et al., 2021); and risdiplam, an oral small-molecule *SMN2* splicing modifier (Darras et al., 2021; Mercuri et al., 2022). These therapies have demonstrated substantial improvements in survival, attainment of motor milestones, and quality of life, particularly when administered early (Finkel et al., 2017; Mercuri et al., 2018; Mendell et al., 2017; Day et al., 2021; Mercuri et al., 2021; Darras et al., 2021; Mercuri et al., 2022). Increasing implementation of prenatal and newborn-screening programs is enabling earlier intervention, when motor neurons are largely preserved, thus potentially improving the durability of outcomes (Mežnarić et al., 2025).

In this review, we summarise current evidence on the real-world effectiveness, safety, and economic aspects of approved SMA therapies, while also outlining emerging treatment strategies and key implications for clinical practice, research, and health policy.

## 2 Methods

This review was conducted as a narrative review with scoping elements to integrate clinical, translational, and pharmacoeconomic evidence regarding currently approved and investigational therapies for SMA. A targeted literature search was performed using PubMed and Scopus databases, supplemented by manual screening of the reference lists of relevant articles. The search included publications from 2015 onwards. The search terms included spinal muscular atrophy, SMA, SMN, *SMN1*, *SMN2*, nusinersen, risdiplam, onasemnogene abeparvovec, disease-modifying therapies, gene therapy, antisense oligonucleotides, real-world evidence, safety, pharmacovigilance, adverse drug reactions, cost-effectiveness, health technology assessment, newborn screening, neuromuscular junction, and investigational therapies.

Studies were included if they provided data on SMA pathophysiology, molecular mechanisms, clinical efficacy, real-world effectiveness, safety, pharmacoeconomic evaluations, treatment switching, combination therapies, biomarkers, or emerging therapeutic strategies. Both experimental and clinical studies, including randomised controlled trials (RCTs), observational studies, registry analyses, pharmacovigilance studies, health economic evaluations, and relevant high-quality reviews, were considered when contributing to the mechanistic understanding, clinical translation, or healthcare policy perspectives in SMA. Exclusion criteria included non-English publications, conference abstracts without full text, and studies not directly related to SMA therapeutics, outcomes, safety, or economic aspects.

Manuscript selection was further refined through expert consensus among the authors based on the relevance, methodological quality, and contribution to the conceptual framework of the review. Evidence was synthesised narratively and organised thematically. As a narrative review, this work does not adhere to formal systematic review methodologies (e.g., PRISMA) and does not include a structured risk-of-bias assessment; therefore, potential selection bias and heterogeneity of included studies should be acknowledged. Nevertheless, this approach is appropriate for synthesising diverse and evolving evidence in a rare disease such as SMA and for providing a comprehensive overview of the current landscape of therapeutics, clinical management, and patient care.

## 3 Efficacy and effectiveness data overview

SMA treatment has advanced substantially with the introduction of disease-modifying therapies such as nusinersen, onasemnogene abeparvovec, and risdiplam. This section examines the clinical efficacy and real-world effectiveness of these therapies, including considerations on combination therapies and treatment switching.

### 3.1 Clinical efficacy

Nusinersen is an intrathecally administered antisense oligonucleotide that modifies *SMN2* splicing to increase full-length SMN protein production. In ENDEAR, symptomatic infants with SMA, mostly with two *SMN2* copies and symptom onset before 6 months of age, were assessed using motor milestone response and event-free survival as key outcomes; nusinersen significantly improved survival and motor milestone achievement, with 51% of treated infants achieving a motor milestone response compared with 0% in the sham-control group (Finkel et al., 2017). In CHERISH, children with later-onset SMA, mainly type 2 disease, showed significant improvement or stabilization in HFMSE scores, with greater benefit in younger patients and those treated earlier in the disease course (Mercuri et al., 2018).

Onasemnogene abeparvovec is a one-time intravenous AAV9-mediated gene replacement therapy that delivers a functional copy of *SMN1*. In the START trial, infants with type 1 SMA and two *SMN2* copies showed markedly improved survival without permanent ventilation and clinically meaningful motor milestone acquisition, including independent sitting in several patients receiving the therapeutic dose (Mendell et al., 2017). The STR1VE studies confirmed these findings in larger symptomatic type 1 SMA cohorts, demonstrating high event-free survival and motor milestone achievement, including independent sitting, with outcomes generally more favourable when treatment was administered earlier (Day et al., 2021; Mercuri et al., 2021). More recently, the intrathecal formulation of onasemnogene abeparvovec received FDA approval for patients aged 2 years and older with SMA, while approval from EMA is under review following a positive Committee for Medicinal Products for Human Use opinion adopted on 23 April 2026 (European Medicines Agency, 2026).

Risdiplam is an orally administered small-molecule *SMN2* splicing modifier that increases endogenous SMN protein production. In FIREFISH, symptomatic infants with type 1 SMA and two *SMN2* copies were assessed for sitting without support and survival without permanent ventilation; risdiplam treatment led to survival and motor milestone gains, including the ability to sit without support in a subset of infants (Darras et al., 2021). In SUNFISH, a broader later-onset population aged 2–25 years with non-ambulant type 2 or 3 SMA showed significant improvement in MFM32 scores compared with placebo, with findings supporting benefit across paediatric, adolescent, and young-adult patients (Mercuri et al., 2022).

Collectively, these pivotal trials established nusinersen, onasemnogene abeparvovec, and risdiplam as disease-modifying therapies that improve survival, motor milestone achievement, or motor function across infantile- and later-onset SMA, with the greatest benefits consistently observed when treatment is initiated early (Finkel et al., 2017; Mercuri et al., 2018; Mendell et al., 2017; Day et al., 2021; Mercuri et al., 2021; Darras et al., 2021; Mercuri et al., 2022).

### 3.2 Real-world effectiveness

While RCTs provide methodologically robust evidence, their strict inclusion criteria often limit generalisability. Thus, real-world evidence has become critical in validating the translational impact of these therapies across broader, more heterogeneous populations (Dayer et al., 2024). Observational studies and registry-based data have demonstrated that nusinersen’s benefits on motor function and survival extend to broader SMA populations, including patients treated at older ages, adults, and individuals with varied baseline motor function or disease severity (Cho et al., 2023; Hagenacker et al., 2020; Maggi et al., 2020). Long-term real-world follow-up generally indicates motor improvement or stabilization, with earlier treatment initiation associated with better outcomes (Cho et al., 2023).

Real-world observational cohorts of onasemnogene abeparvovec have generally confirmed functional improvement and acceptable safety outside trial settings, including in older or heavier children and in national treatment cohorts (Gowda et al., 2024; SMArtCARE and Swiss-Reg-NMD Study Group, 2024; Desguerre et al., 2024). These studies support the effectiveness of gene therapy beyond highly selected pivotal-trial populations, while also reinforcing that earlier treatment is associated with more favourable motor outcomes.

Real-world data for risdiplam remain more limited than that for nusinersen, but they are increasing, particularly in adult and previously treated populations. Available observational studies indicate motor stabilization or improvement in some patients, with the oral route supporting treatment access where intrathecal administration is difficult or undesirable (Sitas et al., 2024).

### 3.3 Combination therapies and treatment switching

Given the variability in disease presentation, progression, and patient-specific factors in SMA, personalised treatment strategies, including treatment switching and combination or sequential therapy, are increasingly being discussed. Clinical trial evidence for combination or sequential use remains limited. Available data are mainly derived from studies evaluating add-on or subsequent treatment after prior disease-modifying therapy, and prospective controlled evidence is still needed to define the optimal timing, patient selection, safety, and efficacy of these approaches (Cheng et al., 2026; Okamoto, 2026; Bekircan-Kurt et al., 2025).

In real-world clinical practice, treatment switching is frequently observed. Patients may transition from nusinersen to risdiplam because of administration-route preference, tolerability, access issues, or perceived suboptimal response. Observational studies indicate that switching is generally feasible and may maintain or improve motor function in selected patients, although the findings are mainly derived from retrospective or uncontrolled cohorts (Belančić et al., 2025a; Cheng et al., 2026; Okamoto, 2026; Bekircan-Kurt et al., 2025).

Sequential or combined use of gene replacement therapy with splice-modifying agents is also being explored, based on the rationale that enhancing SMN restoration through complementary mechanisms may benefit patients with incomplete response to the initial therapy. However, routine use of combination or sequential therapy should await stronger prospective evidence, given the unresolved questions regarding additive benefit, safety, timing, and cost-effectiveness (Cheng et al., 2026; Okamoto, 2026; Bekircan-Kurt et al., 2025).

Overall, treatment switching and combination strategies represent an important but still evolving area of SMA care, requiring prospective studies and harmonised real-world data to guide clinical decision-making.

## 4 Safety data overview

The pivotal clinical trials supporting the approval of SMA therapies—nusinersen (ENDEAR and CHERISH) (Finkel et al., 2017; Mercuri et al., 2018), onasemnogene abeparvovec (START and STR1VE) (Mendell et al., 2017; Day et al., 2021; Mercuri et al., 2021), and risdiplam (FIREFISH and SUNFISH) (Darras et al., 2021; Mercuri et al., 2022)—provided foundational safety data demonstrating acceptable safety profiles within the context of significant clinical benefits. These trials identified adverse drug reactions (ADRs) such as procedural risks for nusinersen, hepatotoxicity for onasemnogene abeparvovec, and gastrointestinal events for risdiplam. However, as these therapies have transitioned to broader clinical use, robust post-marketing safety surveillance has become essential to capture real-world safety signals, particularly for rare and long-term ADRs (Table 1).

**TABLE 1:** Overview of EMA-approved SMA therapies—mechanism, administration, clinical trials, and safety profile.

Medicine name	Type of molecule and mechanism	Route of administration	Key trials leading to approval	EMA approval year	Safety profile
Nusinersen	Antisense oligonucleotide; modulates *SMN2* splicing to increase SMN protein	Intrathecal	ENDEAR, CHERISH	2017	Known ADRs include post-lumbar puncture syndrome, headache, back pain, and procedural risks due to intrathecal delivery. Real-world data confirm high incidence of post-lumbar puncture complications
Onasemnogene abeparvovec	Gene replacement therapy; delivers functional *SMN1* gene via AAV9 vector	Intravenous	START, STR1VE	2020 (conditional), 2022 (full)	Hepatotoxicity is significant; liver function monitoring is recommended. Other ADRs include thrombocytopenia and transient elevated troponin. Real-world data align with regulatory safety warnings
Risdiplam	Oral small-molecule *SMN2* splicing modifier; increases SMN protein	Oral	FIREFISH, SUNFISH	2021	ADRs include gastrointestinal issues (diarrhoea and vomiting) and respiratory events. Real-world data support known ADRs but highlight need for ongoing evaluation

Abbreviations: ADR, adverse drug reaction; EMA, European Medicines Agency; SMN, survival motor neuron.

*The methodology and results of key clinical trials are detailed in the main manuscript text and in other sources (see cited clinical trials for a comprehensive review).

Post-marketing pharmacovigilance utilising spontaneous ADR reports collected in EudraVigilance, complemented by mandated post-authorisation efficacy studies (PAES) and risk management plans (RMPs), has deepened the understanding of the safety profiles of these therapies. Analysis of the most frequently reported preferred terms (PTs) with corresponding reporting odds ratios (RORs) for nusinersen identified ADRs consistent with its intrathecal administration, including vomiting and back pain, along with procedural complications such as post-lumbar puncture syndrome and procedural pain. Importantly, several frequently reported PTs—especially those related to the respiratory system such as respiratory failure, respiratory disorder, pneumonia, and respiratory infections—are likely manifestations of underlying disease rather than true ADRs. Impaired respiratory muscle function, ineffective cough, and scoliosis contribute to an increased risk of pulmonary infections and respiratory failure in early-onset SMA (Belančić et al., 2025b).

Similarly, for onasemnogene abeparvovec, the most frequently reported PTs aligned with known ADRs listed in the EU product information, including pyrexia, vomiting, hepatotoxicity/transaminases increase, elevated troponin I, and thrombocytopenia, reinforcing the necessity for close monitoring of liver function, platelet counts, and cardiac biomarkers following administration. Other reported PTs such as pneumonia and asthenia likely reflect disease complications, though further case-level analysis is warranted (Belančić et al., 2025b). Risdiplam’s frequently reported PTs similarly reflect both known ADRs—such as diarrhoea and urinary-tract infections—and underlying disease symptoms including pneumonia, respiratory failure/disorders, muscular weakness, and respiratory-tract infections. Notably, weight increase observed in this patient population could be a therapeutic benefit rather than an ADR, given that many SMA patients experience undernutrition due to bulbar dysfunction. Reports of asthenia, sepsis, abdominal pain, constipation, and abnormal product taste may represent a mixture of disease complications and potential safety signals. Thus, risdiplam’s association with gastrointestinal and respiratory events, while expected, necessitates further evaluation, particularly concerning its long-term safety (Belančić et al., 2025b).

Monitoring trends in adverse reaction reporting over time can offer insights into the real-world safety of these therapies. When serious adverse event reports consistently decrease while the medication’s utilisation increases, it often indicates a favourable safety profile. Conversely, an increase in such reports may indicate emerging safety concerns that deserve further scrutiny. ADR reporting for nusinersen demonstrated a significant decline 2 years after market entry, which is consistent with the Weber effect and possibly due to decreased utilisation as alternative therapies emerged (Hartnell and Wilson, 2004; Belančić et al., 2025b). In contrast, risdiplam showed a sustained increase in ADR reports, potentially linked to treatment switching and the oral route of administration, which may predispose patients to gastrointestinal events (Zhuang et al., 2023; Belančić et al., 2025b). The persistent increase in respiratory-related ADRs reported with risdiplam calls for continued vigilance to differentiate disease progression from therapy-related effects (Zhuang et al., 2023; Chen et al., 2025; Belančić et al., 2025b).

Because SMA progression and treatment-related events may overlap clinically, ongoing safety evaluation requires complementary post-marketing data from spontaneous reporting systems together with disease registries and longer-term real-world follow-up studies (Balaji et al., 2025). Given the high costs and complex safety profiles of SMA DMTs, enhanced safety monitoring and careful patient selection are essential to optimise their benefit–risk balance (Kirschner et al., 2024; Mishra and Mishra, 2025). Ultimately, these efforts aim to maximise patient outcomes while ensuring efficient healthcare resource utilisation.

## 5 Cost-effectiveness overview

Although DMTs have substantially improved survival and motor outcomes in SMA, their high costs pose major challenges for healthcare systems. Economic evaluations such as cost-effectiveness analyses are essential to inform healthcare resource allocation, pricing decisions, and reimbursement policies for these treatments.

Evidence indicates that nusinersen, risdiplam, and onasemnogene abeparvovec are generally not deemed cost-effective across different settings, particularly when using conventional cost-effectiveness thresholds (Belančić et al., 2025c). An additional systematic review of 20 economic studies published between 2017 and 2022 showed that onasemnogene abeparvovec and risdiplam were regarded as cost-effective relative to nusinersen; however, since nusinersen itself is not cost-effective compared to the best supportive therapy, these comparisons do not support unequivocal recommendations for treatment choice (Motta-Santos et al., 2024).

Onasemnogene abeparvovec, despite a high upfront cost (often exceeding $2 million per patient), yields considerable health benefits, including improved survival and motor milestone achievements in infants, which improves its economic profile when compared with extended nusinersen treatment costs (Tice et al., 2025). Nonetheless, the incremental cost-effectiveness ratio (ICER) estimates frequently surpass accepted willingness-to-pay (WTP) thresholds due to the substantial drug-acquisition cost. The single-dose administration contrasts with lifelong intrathecal administration of nusinersen, which incurs ongoing procedural costs and risk (Tice et al., 2025). Although onasemnogene abeparvovec is associated with liver injury events, most reported cases are managed with corticosteroid treatment and monitoring; however, severe cases have been described, and their management may require additional healthcare resources (Belančić et al., 2025d).

Nusinersen’s cost-effectiveness estimates are generally unfavourable, primarily due to its high acquisition cost and the need for repeated intrathecal maintenance dosing every 4 months over a lifetime (Motta-Santos et al., 2024; Belančić et al., 2025c). This imposes considerable cumulative costs and procedural burden. Some models reported nusinersen ICERs exceeding $1 million per quality adjusted life year (QALY) gained compared with the best supportive care, showing limited economic appeal despite clear clinical efficacy (Motta-Santos et al., 2024; Zuluaga-Sanchez et al., 2019; Belančić et al., 2025c).

Risdiplam has a different cost structure from both onasemnogene abeparvovec and nusinersen, with economic results varying according to the comparator choice, treatment duration, and model assumptions (Motta-Santos et al., 2024; Belančić et al., 2025c). Cost-effectiveness analyses indicate that risdiplam’s ICERs are generally more favourable than that of nusinersen but variable when compared with that of onasemnogene abeparvovec; limited direct comparative clinical trial data introduce uncertainty. Real-world evidence on long-term efficacy and safety is emerging but incomplete, constraining definitive economic conclusions (Motta-Santos et al., 2024; Yousefi et al., 2025; Belančić et al., 2025c). Table 2 includes global pharmacoeconomic evaluations of SMA treatments. Across the published studies, the dominant pattern is that current SMA therapies are usually not cost-effective against standard/best supportive care or local willingness-to-pay thresholds at list prices. The main exception is in some US head-to-head models comparing onasemnogene with long-term repeated nusinersen treatment, where onasemnogene appears cost-effective or even dominant; however, Dutch, Australian, Brazilian, and Thai analyses still show large ICERs or non-cost-effectiveness, highlighting how strongly the results depend on the drug price, assumed durability of benefit, utility inputs, and comparator choice (Zuluaga-Sanchez et al., 2019; Thokala et al., 2020; Malone et al., 2019; Dean et al., 2021; Broekhoff et al., 2021; Wang et al., 2022; Fernandes et al., 2024; Khuntha et al., 2025).

**TABLE 2:** Pharmacoeconomic analyses assessing SMA treatments.

Study	Setting/population	Interventions and comparators	Main economic result	Authors’ conclusion
Zuluaga-Sanchez et al., 2019	Sweden; infantile-onset and later-onset SMA	Nusinersen vs. standard of care	ICERs including caregiver QALYs: 5.64 million SEK/QALY (infantile-onset) and 3.19 million SEK/QALY (later-onset)	Not cost-effective at the cited 2 million SEK/QALY rare-disease threshold
Thokala et al., 2020	United States; infantile-onset SMA	Nusinersen vs. best supportive care	Base-case ICER approximately US$1.112 million/QALY; scenario/sensitivity analyses did not reduce the ICER below US$990,000/QALY	Exceeded traditional US cost-effectiveness thresholds
Malone et al., 2019	United States; SMA type 1 infants	Onasemnogene abeparvovec (AVXS-101) vs. nusinersen	With assumed onasemnogene price range US$2.5–US$5.0 million, ICER ranged from –US$203,072 to US$31,379/QALY vs. nusinersen	Onasemnogene was judged cost-effective vs. chronic nusinersen in this model
Dean et al., 2021	United States; symptomatic SMA type 1 in children <2 years	Onasemnogene abeparvovec vs. nusinersen vs. best supportive care	Onasemnogene was dominant vs. nusinersen and US$161,648/QALY vs. best supportive care	Cost-effective vs. nusinersen at list price US$2.125 million but above common US thresholds vs. best supportive care
Broekhoff et al., 2021	Netherlands; SMA type 1	Onasemnogene abeparvovec vs. nusinersen vs. best supportive care	Base-case ICERs: €138,875/QALY vs. best supportive care and €53,447/QALY vs. nusinersen; relapse within 10 years could increase the ICER up to 6-fold	Unlikely to be cost-effective under Dutch reference values; price would need to fall to approximately €680,000
Wang et al., 2022	Australia; SMA type 1	Onasemnogene abeparvovec and nusinersen vs. standard of care; onasemnogene vs. nusinersen	ICERs: A$1,808,471/QALY for onasemnogene vs. standard care, A$2,772,798/QALY for nusinersen vs. standard care, and A$1,238,288/QALY for onasemnogene vs. nusinersen	Neither therapy was cost-effective at A$50,000/QALY
Fernandes et al., 2024	Brazil; SMA type 1	Onasemnogene abeparvovec vs. nusinersen and risdiplam	Versus nusinersen, onasemnogene added approximately 3 QALYs at an incremental cost of R$2,468,448, giving an ICER of R$742,890.92/QALY; risdiplam showed extended dominance	Onasemnogene appeared more effective, but with substantial incremental cost
Khuntha et al., 2025	Thailand; symptomatic SMA type 1 and types 2–3	For type 1: Onasemnogene or risdiplam vs. standard care; for types 2–3: Risdiplam vs. standard care	Type 1 ICERs: US$163,102/QALY (onasemnogene) and US$158,357/QALY (risdiplam); types 2–3 risdiplam: US$496,704/QALY	None of the evaluated options were cost-effective at the Thai threshold

Abbreviations: ICER, incremental cost-effectiveness ratio; QALY, quality-adjusted life-year; SMA, spinal muscular atrophy.

Onasemnogene abeparvovec is also available as an intrathecal formulation, Itvisma®/onasemnogene abeparvovec-brve, in some countries for patients aged ≥2 years with SMA. This should be distinguished from the intravenous formulation evaluated in most pharmacoeconomic studies included in this table (U.S., food and drug administration, 2025; European Medicines Agency, 2026).

Early treatment initiation, especially pre-symptomatic treatment identified via newborn screening, substantially improves clinical outcomes and cost-effectiveness across all therapies. Early intervention enables patients to achieve critical motor milestones, leading to lower healthcare resource use, reduced mortality, and improved quality of life. Economic models incorporating pre-symptomatic treatment scenarios show improved ICERs relative to symptomatic treatment initiation, supporting newborn-screening policy adoption (Tice et al., 2025).

Key model drivers in CEAs include drug-acquisition costs, utility gains linked to motor milestone attainment, survival benefits, and treatment-related adverse events. Uncertainty remains regarding long-term durability of treatment effects, especially for onasemnogene abeparvovec and risdiplam, as clinical experience expands. The rarity of SMA limits sample sizes for robust longitudinal data, and modelling of natural history involves assumptions that influence incremental effectiveness estimates (Tice et al., 2025).

Finally, health technology assessment (HTA) bodies recognise the limitations of traditional cost-effectiveness thresholds when evaluating rare, severe paediatric disorders such as SMA. Conventional cost-effectiveness analyses tend to assume large patient populations to achieve statistical robustness and economies of scale, yet orphan drugs for SMA are associated with a significantly low prevalence, yielding sparse RW data, high parametric uncertainty, and high per-patient costs that routinely exceed conventional thresholds. Moreover, the WTP thresholds tend to vary significantly across regions, with vast differences being reported between Europe and low- or middle-income countries, while no national threshold has been set in the United States of America. In addition, conventional cost-effectiveness analyses inadequately capture heterogeneous disease trajectories, long-term survival gains from single-dose therapies, and non-quantifiable benefits such as preserved QoL or caregiver burden alleviation. Researchers and experts advocate for adjusted or higher thresholds considering the disease burden, rarity, lack of alternatives, and societal values including caregiver burden. Innovative reimbursement strategies such as outcome-based managed entry agreements and staggered payment models are becoming increasingly relevant to balance affordability and access while managing financial risk during evidence generation (Belančić et al., 2025c; Tice et al., 2025).

In conclusion, while nusinersen, risdiplam, and onasemnogene abeparvovec provide significant clinical benefits for SMA patients, current pharmacoeconomic evidence indicates they are not cost-effective under standard WTP thresholds compared to the best supportive care. Onasemnogene abeparvovec shows relative economic advantage versus nusinersen given its single-dose administration and durable clinical effects, although its high upfront price remains a challenge. Risdiplam’s oral administration and emerging cost-effectiveness signals offer an alternative, but evidence gaps persist. Early treatment initiation is crucial for optimising both clinical and economic outcomes. Further long-term data collection and consideration of modified economic evaluation frameworks are imperative to guide sustainable resource allocation and access policies in SMA care.

## 6 Medicines under investigation

While first-generation SMA therapeutics have transformed disease prognosis, their limitations continue to shape the future direction of therapeutic innovation (Figure 1). One important area of progress is through pharmacokinetic and pharmacodynamic optimisation. For example, age-adapted pharmacokinetic modelling and neonatal-specific liquid formulations of risdiplam are being tested in the PUPFISH trial (NCT05808764) to ensure safe and effective SMN induction during the earliest treatment window. Meanwhile, higher-doses nusinersen regimens were tested in the DEVOTE study (NCT04089566) using pharmacodynamic modelling to adjust loading schedules, with the aim of determining whether enhanced intrathecal exposure can improve motor outcomes in older or previously treated patients. In parallel, innovations in delivery technology are beginning to mitigate the operational and pharmacokinetic constraints of current treatments. A clinical trial is being conducted on an implantable system manufactured by Biogen that uses a subcutaneous titanium port connected to a flexible intrathecal catheter (NCT06555419). This system enables repeated dosing without the need for lumbar puncture, potentially improving dosing consistency and reducing procedural risk and complicity, especially for patients with severe scoliosis or spinal fusion.

**FIGURE 1:**
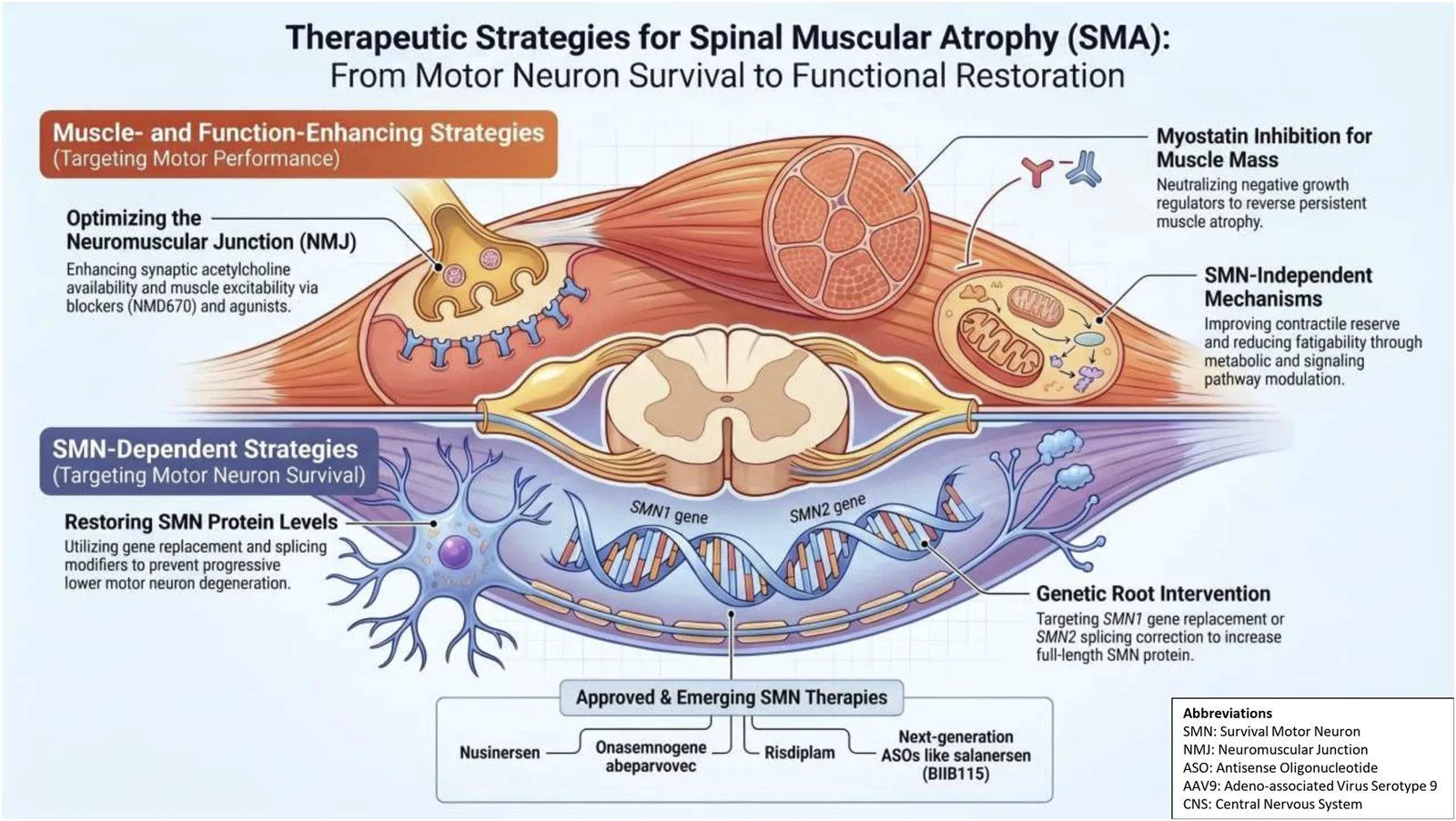
General principle of SMN-dependent and muscle- and function-enhancing therapeutic strategies for SMA.

While these refinements enhance the efficacy and safety of current therapies, they do not resolve the more fundamental limitations of these treatments. For instance, nusinersen distributes poorly beyond the central nervous system (CNS); onasemnogene abeparvovec is constrained by age and weight cut-offs, hepatotoxicity, and uncertain long-term transgene durability; whereas risdiplam, though systemic, achieves only modest SMN induction. Importantly, it is increasingly evident that many key aspects of SMA pathophysiology, including impaired neuromuscular junction structure, reduced muscle strength, fatigability, mitochondrial stress, and abnormal intercellular signalling, cannot be reverted solely by the restoration of SMN expression (Torri et al., 2024).

These limitations have motivated a growing pipeline of next-generation therapies (Moultrie et al., 2025; Weng et al., 2025). Advances in nucleic-acid engineering have produced antisense oligonucleotides with greater stability, higher target affinity, and improved CNS biodistribution, enabling more efficient and durable correction of *SMN2* splicing (Sergeeva et al., 2022). For example, BIIB115 (salanersen) uses 2′-O-[2-(methylamino)-2-oxoethyl] (2′-O-NMA) modification that improves nuclease resistance, increases cellular uptake, and prolongs intraneuronal retention compared to nusinersen (Ling et al., 2025). As a result, salanersen is expected to achieve higher and more durable SMN protein restoration while potentially requiring fewer intrathecal administrations (NCT05575011 and NCT07221669).

Limitations of the first-generation AAV9 product, onasemnogene abeparvovec, are addressed through refinements to vector design. Several next-generation vectors, such as GC101 (NCT05824169 and NCT05901987), GCB-001 (NCT06772402), vesemnogene lantuparvovec (NCT04262804), ANB-004 (NCT05747261), and EXG001-307 (Song et al., 2025), are currently under clinical trial. These agents incorporate optimised promoter systems and codon-enhanced transgenes for higher translational efficiency and capsid-tropism modifications designed to improve CNS specificity (Di Minno et al., 2024). Furthermore, some of these new vectors, such as GC101, are developed for single-dose intrathecal administration, in contrast to onasemnogene abeparvovec, which is delivered intravenously and, therefore, requires very high systemic AAV9 doses associated with hepatic toxicity and strict age- and weight-based eligibility limits. The single-dose intrathecal administration also overcomes the procedural burden of nusinersen, which requires repeated intrathecal procedures throughout life.

Emerging genetic and epigenetic approaches aim to complement SMN-restoring therapies (Chongmelaxme et al., 2025). Small-molecule inhibitors of protein methyltransferases such as PRMTs promote exon-7 inclusion in *SMN2* pre-mRNA and act synergistically with nusinersen to enhance SMN production in preclinical mouse models (Guerra et al., 2023). In parallel, preclinical CRISPR/Cas9 systems have successfully increased SMN protein levels, improved motor function, and extended survival in SMA mice, indicating the potential for a future one-time, curative therapeutic modality (Hatanaka et al., 2024).

An emerging group of therapies focuses on neuromuscular transmission. These therapies have been used sparingly as adjunctive symptomatic treatments in selected patients with SMA, but they are not commonly used disease-modifying therapies and should be distinguished from the newer investigational agents currently under development. Pyridostigmine, an acetylcholinesterase inhibitor, has shown modest functional benefit by prolonging synaptic acetylcholine availability and partially compensating for transmission failure (Stam et al., 2022). β_2_-Adrenergic agonists such as salbutamol, widely used in neuromuscular disorders, may stabilise the neuromuscular junction structure and enhance contractile performance (Xing et al., 2025). Meanwhile, NMD670, a selective ClC-1 chloride-channel blocker, enhances muscle excitability by reducing membrane hyperpolarisation and improving the muscle response to motor-neuron input (Skov et al., 2024; Ruijs et al., 2025). Furthermore, the therapeutic effects of sodium-channel modulation (e.g., mexiletine) and β_2_-adrenergic stimulation (e.g., clenbuterol) observed in SBMA and other neuromuscular disorders indicate that the targeted modulation of muscle excitability and neuromuscular transmission may complement SMN restoration in SMA (Martínez-Rojas et al., 2021; Yamada et al., 2022).

Another major line of development focuses on the intrinsic muscle pathology of SMA. Even with SMN restoration, many patients exhibit persistent muscle atrophy, impaired hypertrophic signalling, and reduced contractile reserve, indicating that muscle itself is a critical therapeutic target (Zeng et al., 2025). Myostatin, a potent negative regulator of skeletal-muscle growth, has, therefore, become a key node for intervention (Abati et al., 2022; Wetzlich et al., 2025). SRK-015 (apitegromab), a monoclonal antibody designed to bind and neutralise promyostatin and latent myostatin with high structural specificity (Dagbay et al., 2020), showed a statistically significant improvement in motor outcomes in the phase-3 SAPPHIRE trial, but the observed effect size was small, and the clinical relevance of this change should be interpreted cautiously (Crawford et al., 2025). Similar strategies are being evaluated with RO7204239, an anti-myostatin agent currently being tested in combination with risdiplam in the MANATEE study, and with taldefgrobep alfa, a recombinant myostatin inhibitor under investigation in the RESILIENT trial (Servais et al., 2024a). Together, these agents represent an SMN-independent strategy that may substantially augment the functional gains achieved with existing disease-modifying treatments.

Exosome-based therapeutics represent another potential approach for SMA therapy (Fajkić et al., 2025). Extracellular vesicles can carry SMN protein and full-length SMN transcripts (Nash et al., 2017, Trifunov et al., 2023), and engineered exosomes in other neurological models have successfully delivered antisense oligonucleotides and neurotrophic factors to target cells (Adamus et al., 2021; Putthanbut et al., 2024; Fu et al., 2025). These findings indicate that exosome-based carriers could, in principle, be adapted to deliver SMN-restoring or neuromuscular-junction-supportive cargos in SMA. Preclinical studies show that SMN-enriched exosomes can restore nuclear body formation in SMN-deficient cells, while stem-cell-derived exosomes can enhance motor-neuron survival and reduce apoptosis independently of SMN (Virla et al., 2024; René and Parks, 2025). Additional strategies, such as delivering pro-synaptic miRNAs, Agrin fragments, GDNF, or IGF-1 via exosomes (Ma et al., 2019; Maggio et al., 2019; Chen et al., 2020; Agostini et al., 2025), offer a biologically natural means of stabilising the neuromuscular junction structure and strengthening neuromuscular signalling.

Recent multi-omics studies have deepened our understanding of SMA as a complex, multisystem network disorder rather than a purely motor-neuron disease, revealing widespread dysregulation across RNA processing, translation, cytoskeletal organisation, metabolism, signalling pathways, and cellular stress responses (Tapken et al., 2025). These systems-level insights are beginning to define a broadened therapeutic landscape in which SMN restoration is necessary but insufficient and where diverse molecular pathways, ranging from kinase signalling and metabolic homeostasis to proteostasis, neurotrophin pathways, and neuromuscular junction (NMJ) maintenance, emerge as rational drug targets. As the molecular architecture of SMA becomes clearer, it reveals multiple, tractable intervention points that are likely to shape the next-generation of treatments aimed at true neuromuscular restoration. The rapidly evolving pipeline reflects how multidisciplinary innovations, from molecular engineering and vector design to neuromuscular physiology, systems pharmacology, and regenerative biology, are reshaping SMA therapeutics, marking a shift from survival-focused medicine toward comprehensive, lifelong restoration of neuromuscular functions.

## 7 Discussion

SMA research and clinical management continue to evolve at a rapid pace, driven by remarkable therapeutic advances over the last decade. Addressing the current challenges in SMA treatment and care requires a coordinated strategy that extends beyond therapeutic innovation alone. Although DMTs have transformed outcomes, their exceptionally high costs continue to strain patients, payers, and health systems, while disparities in reimbursement, specialist availability, and geographic access limit equitable uptake. At the same time, important uncertainties remain regarding long-term safety, durability of benefit, and optimal sequencing or combination of therapies, particularly as treated individuals survive longer and present with evolving clinical needs. These issues are compounded by ethical concerns related to fair allocation of scarce resources, unequal access across and within countries, and decision-making in pre-symptomatic or very young patients when long-term outcomes are still being defined. Health system constraints, including fragmented care pathways, inadequate newborn-screening coverage, limited rehabilitation and respiratory support services, and insufficient long-term follow-up infrastructure, further impede comprehensive care delivery, Accordingly, sustainable progress in SMA will depend on policies that promote affordable pricing and reimbursement, broader access to early diagnosis and multidisciplinary care, robust post-marketing surveillance, and ethically grounded, evidence-based frameworks for treatment prioritisation and lifelong support (Moultrie et al., 2025; Groen and Wadman, 2025; Abati et al., 2022; SMA Europe, 2024).

Future directions in SMA focus on optimising clinical outcomes through earlier diagnosis and treatment, improving dosing regimens, expanding combination approaches, and innovating novel therapies addressing both motor neuron survival and muscle preservation. In parallel, advances in biomarkers, rehabilitative care, and health services aim to maximise the quality of life for patients with diverse SMA phenotypes (Moultrie et al., 2025; Groen and Wadman, 2025).

A major frontier is the further push toward pre-symptomatic and even prenatal treatment initiation. Newborn screening programs enable rapid identification of infants with the genetic diagnosis of SMA before symptom onset, allowing immediate therapeutic intervention that is critical to preventing irreversible motor neuron loss (Cooper et al., 2024; Vrščaj et al., 2024; Mežnarić et al., 2025). Clinical trials such as RAINBOWFISH have demonstrated that early administration of risdiplam in neonates yields higher rates of independent sitting and walking compared to symptomatic treatment initiation, defining a new standard of care (Servais et al., 2024b). Going further, research into prenatal administration of SMA therapies, including direct *in utero* delivery of gene or splicing-modulator agents, is underway, with promising early safety and efficacy signals. These efforts raise the prospect of normal or near-normal motor development and highlight the critical window of opportunity in early life for altering disease trajectories (Tizzano and Finkel, 2017).

Furthermore, optimising the dosing and delivery of existing therapies represents another area of focus. For example, nusinersen dosing regimens are being refined to balance maximal therapeutic benefit with minimal invasiveness and side effects, such as post-lumbar puncture complications. Higher doses and extended-interval regimens are being evaluated in ongoing trials (Takahashi et al., 2025). Similarly, novel formulations and delivery routes for onasemnogene abeparvovec, including intrathecal administration, aim to improve accessibility and applicability in older or heavier patients while reducing systemic exposure risks (Kichula et al., 2021). Oral agents such as risdiplam continue to be studied for long-term effectiveness and combination potential with other treatments to enhance synergistic effects on both neuronal and muscular targets (Kokaliaris et al., 2024; Weng et al., 2024).

In addition, combination therapy approaches present one of the most exciting future avenues. Given the complex SMA pathophysiology involving both motor neuron loss and the resultant muscle atrophy and weakness, strategies that simultaneously enhance SMN protein levels and stimulate muscle growth or repair are under active investigation. Agents such as apitegromab, a myostatin inhibitor, and taldefgrobep alfa, a muscle-targeting antibody, are currently in clinical development alongside standard SMN-modifying treatments (Abati et al., 2022; SMA Europe, 2024; Moultrie et al., 2025). Early-phase trials signal improvements in muscle strength and functional outcomes beyond those seen with SMN augmentation alone. The integrative use of gene therapy, antisense oligonucleotides, and muscular enhancers is hypothesised to confer additive or synergistic benefits, potentially changing long-term prognosis (Abati et al., 2022; SMA Europe, 2024; Moultrie et al., 2025).

Parallel to improving pharmacological modalities, advances in biomarkers for SMA are expected to enhance individualised patient monitoring and therapeutic decision-making. Quantitative biomarkers such as serum neurofilament levels, SMN protein quantification, and electrophysiological measures hold promise for assessing disease progression and response to treatment with greater sensitivity and timeliness than traditional clinical scales (Corti et al., 2025; Gao et al., 2025; Yan et al., 2025). Biomarker-guided treatment personalisation could enable early switching or combining therapies for suboptimal responders and reduce overtreatment in stable patients.

Moreover, innovations in rehabilitative and supportive care are concurrently vital for future SMA management paradigms. State-of-the-art physiotherapy techniques, orthotic interventions, and respiratory and nutritional support models continue to improve functional independence and quality of life (Song et al., 2025; Sun et al., 2025; Shin, 2024; Song and Ke, 2025). Integration of multidisciplinary care teams for holistic management tailored to changing patient needs will be essential as therapeutic outcomes improve life expectancy and alter disease phenotypes. Digital health technologies and telemedicine solutions are gaining traction in facilitating remote monitoring, timely intervention, and patient engagement (Song et al., 2025; Sun et al., 2025; Shin, 2024; Song and Ke, 2025).

From a research infrastructure perspective, international collaborations and large patient registries will continue to play a critical role in advancing SMA knowledge. Sharing real-world effectiveness data and long-term safety outcomes across diverse patient populations will inform the best practices and accelerate development of next-generation therapies. Emerging gene-editing technologies such as CRISPR/Cas9 offer a promising future possibility for curative correction of *SMN1* mutations, although substantial challenges remain before clinical application (Balaji et al., 2025).

Finally, the economic and access landscape for SMA treatments will influence future directions in the field. With substantial treatment costs posing barriers globally, health technology assessments and policy frameworks are increasingly emphasising value-based pricing, early-access programs, and equity in care delivery. Thus, continued advocacy and research into cost-effectiveness alongside clinical innovation will be crucial to ensure that breakthroughs translate into improved outcomes broadly (Tice et al., 2025; Elshahawy et al., 2025; Al-Jedai et al., 2025).

In summary, the future of SMA lies in pushing therapeutic boundaries through earlier and more precise interventions, multi-modal combination strategies, biomarker integration, and comprehensive supportive care, all facilitated by robust global cooperation. These advances promise to transform SMA from a devastating neurodegenerative disorder into a manageable, potentially preventable condition with improved life quality and expectancy for affected individuals.

The extraordinary advances in SMA research and treatment over the past decade have not only transformed outcomes for patients but also offer important lessons and strategies broadly applicable to the wider field of rare diseases. With over 7,000 rare diseases collectively affecting millions worldwide, the SMA experience provides a valuable model for accelerating therapeutic development, optimising clinical care pathways, and addressing challenges unique to rare-disease management.

The deeper lesson of SMA is that the therapeutic breakthroughs in this setting were only possible through decades of research, from the discovery of *SMN1* and the modifier gene *SMN2* to detailed biochemical characterisation of the SMN complex, spliceosomal snRNP assembly, and the organisation of nuclear bodies. Cell-biological and physiological studies defining SMN’s roles in RNA trafficking, axonal maintenance, cytoskeletal regulation, mitochondrial homeostasis, and neuromuscular-junction maturation provided the mechanistic blueprint that made targeted therapies possible (Hong et al., 2024; Dawood et al., 2025). This trajectory illustrates a universal principle in rare-disease medicine: transformative treatments emerge not from clinical trials alone but from deep mechanistic understanding of gene function, cellular pathology, and systemic physiology. SMA serves as a model showing that for rare diseases broadly, the path to curative therapies begins with identifying the causal gene, elucidating its function with biochemical precision, and mapping the downstream pathways that can be rationally targeted—the foundation upon which all subsequent translational success is built (Hong et al., 2024; Dawood et al., 2025). Recent advances in genomic technologies are markedly accelerating this approach, allowing the etiological understanding and therapeutic innovations of other rare diseases.

One of the most significant lessons from SMA is the critical role of early diagnosis and intervention. The implementation of widespread newborn-screening programs for SMA has facilitated the identification of affected infants before symptoms arise, enabling timely treatment initiation that markedly improves prognosis (Wong et al., 2024; Wang et al., 2025). This paradigm shift towards pre-symptomatic diagnosis and treatment underscores the transformative potential of early detection, which, if applied to other rare diseases with known genetic aetiologies and effective therapies, could similarly alter disease trajectories and improve clinical outcomes. Consequently, the SMA experience highlights the value of incorporating rare diseases into newborn-screening panels and expanding genomic testing infrastructure as a cornerstone of rare-disease care (Jiang et al., 2023; Tariq et al., 2025).

The development and regulatory approval of multiple disease-modifying treatments for SMA within a relatively short timeframe exemplify the potential of collaborative translational research and innovative clinical trial designs in rare diseases. SMA’s success story involved robust partnerships among academic centres, industry, patient advocacy organisations, and regulatory agencies to rapidly identify therapeutic targets, develop diverse modalities, including gene therapy, antisense oligonucleotides, and oral splicing modifiers, and conduct pivotal trials despite small patient populations (Chaytow et al., 2021). This multidisciplinary, patient-engaged approach demonstrates how concerted scientific and community efforts can overcome inherent challenges in rare-disease drug development, such as limited natural history data and ethical considerations in placebo-controlled trials. The SMA model encourages adoption of adaptive trial designs, real-world evidence collection, and patient-reported outcomes to maximise data acquisition and regulatory readiness across other rare diseases (Chaytow et al., 2021).

Another important lesson lies in the nuanced understanding of disease heterogeneity and the consequent tailoring of therapeutic strategies. SMA encompasses a clinical spectrum ranging from severe infantile-onset to milder adult-onset forms, with variability influenced by genetic modifiers such as *SMN2* copy number. This heterogeneity required the development of multiple therapeutic approaches adapted to different phenotypes and stages, including individualised dosing regimens and treatment combinations (Farrar and Kiernan, 2015). Such personalised medicine principles are highly relevant in other rare diseases exhibiting phenotypic diversity and complex pathophysiology, emphasising the need for biomarker development, genotype–phenotype correlation studies, and flexible treatment paradigms (Farrar and Kiernan, 2015).

The SMA field also provides insights into the importance of multidisciplinary care delivery and integration of supportive therapies. Even with effective disease-modifying treatments, patients benefit from comprehensive management including physical therapy, respiratory support, nutritional interventions, orthopaedic care, and psychosocial services to optimise function and quality of life (Spinal Muscular Atrophy Care Guidelines, 2018; SMA Europe, 2017). This holistic care model, coordinated by expert multidisciplinary teams, should be a blueprint for rare-disease care more broadly, particularly where progressive disability and complex multi-organ involvement are common. Moreover, the incorporation of telemedicine and digital health tools in SMA care offers scalable solutions to overcome geographical and resource barriers often encountered by rare-disease patients, informing future healthcare delivery innovations (Peterson et al., 2024).

SMA research has also highlighted the growing importance of patient advocacy groups and community engagement in driving research agendas, raising awareness, and supporting patients and families. Organisations such as Cure SMA have been instrumental in funding research, facilitating clinical trial recruitment, and influencing policy and reimbursement decisions. This active involvement creates a powerful feedback loop that accelerates translational progress and ensures that research remains patient-centred—a model that can be readily emulated in other rare-disease communities (Balaji et al., 2025; Peterson et al., 2022).

Furthermore, the SMA experience underlines the critical role of HTA, economic evaluation, and pricing strategies in ensuring patient access to high-cost rare-disease therapies. SMA treatments such as gene therapy and antisense oligonucleotides come with substantial financial costs, necessitating innovative reimbursement models and value-based pricing agreements to balance innovation incentives with healthcare sustainability (Belančić et al., 2025c; Paracha et al., 2022; Elshahawy et al., 2025). Strategies developed to address these challenges in SMA, including outcome-based agreements and early-access programs, offer valuable frameworks for other rare diseases facing similar economic pressures (Belančić et al., 2025c; Paracha et al., 2022; Elshahawy et al., 2025).

Finally, the recent emergence of next-generation therapeutic technologies in SMA, such as gene editing and combination treatment regimens, foreshadows broader applications in rare diseases. Advances in precision genome-editing tools such as CRISPR/Cas9 hold promise for curative approaches by correcting pathogenic mutations at their source, a concept broadly applicable to monogenic rare diseases (Hatanaka et al., 2024). Additionally, multimodal therapeutic regimens addressing complex disease biology beyond single targets may become increasingly necessary in multifactorial rare conditions, inspired by the combination approaches under exploration in SMA (Proud et al., 2023; Bemanalizadeh et al., 2025).

In conclusion, the journey of SMA research and clinical care offers a comprehensive template from which lessons can be extrapolated to catalyse progress in the rare-disease field. Early diagnosis and treatment, collaborative research infrastructures, tailored and multidisciplinary care, patient advocacy empowerment, economic innovation, and next-generation therapeutic technologies comprise key pillars established in SMA that hold promise for improving outcomes across the diverse spectrum of rare diseases. Embracing these lessons will be vital to unlocking new horizons for millions affected by rare disorders worldwide.

Transformative advances in genetics, newborn screening, and SMN-restoring therapies have reshaped SMA from a fatal infantile disorder into a treatable, chronically evolving condition. Yet, residual motor impairment, heterogeneous response trajectories, and unresolved long-term safety and durability questions highlight the limits of SMN augmentation alone. Integrating emerging SMN-independent strategies with refined biomarkers, earlier diagnosis, and coordinated multidisciplinary care will be central to closing this therapeutic gap. As SMA enters a new biological and clinical era, its trajectory offers a compelling blueprint for accelerating precision therapeutics across rare neurogenetic diseases.
